# Nongenomic oestrogen signalling in oestrogen receptor negative breast cancer cells: a role for the angiotensin II receptor AT1

**DOI:** 10.1186/bcr1509

**Published:** 2006-06-28

**Authors:** Kheng Tian Lim, Niamh Cosgrave, Arnold David Hill, Leonie S Young

**Affiliations:** 1 School of Medicine and Medical Science, St. Vincent's University Hospital, Elm Park, Dublin, Ireland; 2 School of Medicine and Medical Science, UCD Conway Institute of Biomolecular and Biomedical Research, UCD Conway Institute, University College Dublin, Dublin, Ireland

## Abstract

**Introduction:**

Oestrogens can mediate some of their cell survival properties through a nongenomic mechanism that involves the mitogen-activated protein kinase (MAPK) pathway. The mechanism of this rapid signalling and its dependence on a membrane bound oestrogen receptor (ER), however, remains controversial. The role of G-protein-coupled receptor and epidermal growth factor (EGF) receptor in an ER-independent signalling pathway modulated by oestrogen was investigated.

**Methods:**

ER-positive and ER-negative breast cancer cell lines (MCF-7 and SKBR3) and primary breast cancer cell cultures were used in this study. Cell proliferation was assessed using standard MTT assays. Protein and cAMP levels were detected by Western blotting and ELISA, respectively. Antigen localization was performed by immunocytochemistry, immunohistochemistry and immunofluorescence. Protein knockdown was achieved using small interfering RNA technologies.

**Results:**

EGF and oestrogen, alone and in combination, induced cell proliferation and phosphorylation of MAPK proteins Raf and ERK (extracellular signal regulated kinase)1/2 in both ER-negative SKBR3 and ER-positive MCF-7 human breast cancer cell lines. Increased Raf phosphorylation was also observed in primary human breast cultures derived from ER-positive and ER-negative breast tumours. Oestrogen induced an increase in intracellular cAMP in ER-negative SKBR3 human breast cancer cells. Oestrogen-mediated cell growth and phosphorylation of MAPK was modified by the EGF receptor antagonist AG1478, the G-protein antagonist pertussis toxin, and the angiotensin II receptor antagonist saralasin. Knockdown of angiotensin II type 1 receptor (AT1) protein expression with small interfering RNA attenuated oestrogen-induced Raf phosphorylation in ER-negative cells. AT1 receptor was found to be expressed in the cell membrane of breast tumour epithelial cells.

**Conclusion:**

These findings provide evidence that, in breast cancer cells, oestrogen can signal through AT1 to activate early cell survival mechanisms in an ER-independent manner.

## Introduction

Oestrogens induce diverse physiological effects that allow normal development and growth of female reproductive tissues, and regulation of bone integrity, cardiovascular function and the central nervous system. Aberrant expression of oestrogen can induce pathophysiological effects that give rise to the growth of tumours, in particular those of the breast. Classically, the mechanism of action of oestrogen was singularly attributed to the binding of nuclear oestrogen receptor (ER) and subsequently activation of target genes over the course of several hours. More recently, it has become clear that oestrogen may rapidly act on cells in seconds to minutes, implicating a nongenomic mechanism of oestrogen signalling.

In addition to its ability to promote ER-dependent gene transcription, oestrogen rapidly triggers a variety of second messenger signalling events, including mobilization of intracellular calcium [1-3], production of cAMP [4,5], generation of inositol triphosphate [6], and activation of mitogen-activated protein kinase (MAPK) [7-9], phosphatidylinositol 3-OH kinase and AKT/protein kinase B [10-12]. Nongenomic effects of oestrogen purportedly result from the steroid binding a receptor protein in the cell membrane [13].

Membrane ERs have been shown to exist in discrete caveolar domains in the plasma membrane [14,15]. Studies in CHO cells have identified similarly sized nuclear and membrane ER proteins that result from the expression of a single cDNA [16]. Membrane ER is thought to be G-protein linked, with oestrogen binding resulting in activation of many signal transduction pathways that emanate from G protein activation (for extensive review, see [17]). It was recently reported that the E-domain of membrane ER is required for activation of the MAPK cascade [15] and that serine at amino acid 522 is necessary for the translocation of ER-α to the plasma membrane [18]. In breast cancer cells plasma ER is thought to exist as functional dimers when bound by a steroid ligand [19], but oestrogen-dependent endothelial nitric oxide synthase activation in ER-transfected COS cells may not require dimerization [20].

Studies using ER-negative cell lines suggest that oestrogen may also function in an ER-independent manner. Studies from several laboratories have demonstrated that, in ER-negative cells, oestrogen can signal through the G-protein-coupled receptor (GPCR) GPR30 to transactivate epidermal growth factor receptor (EGFR) and activate the MAPK cascade [21,22]. This oestrogen transactivation of EGFR has been shown to be via the release of surface-associated heparin-binding epidermal growth factor [23]. It has been demonstrated that this GPR30-dependent oestrogen induction of MAPK is transient and under the control of a cAMP-dependent negative feedback loop.

Data from the above studies suggest that oestrogen can initiate rapid MAPK signalling in an ER-dependent and ER-independent manner. First, oestrogen can bind a membrane ER, similar or identical to the nuclear receptor, and subsequently activate G proteins; secondly, oestrogen can also directly activate GPCR in the membrane in an ER-independent manner, thereby effecting G protein activation.

More than one GPCR may participate in rapid oestrogen signalling, and it is likely that further complexity in oestrogen-mediated GPCR signalling may occur as a result of coupling of different G protein heterodimers with the same receptor. Angiotensin II receptor is of particular interest as a candidate, oestrogen-interacting GPCR. Inwang and colleagues [24] demonstrated expression of angiotensin II type 1 (AT1) receptors in both normal and diseased human breast tissues. Other studies showed that activation of AT1 receptor stimulates growth factor pathways such as tyrosine kinase phosphorylation and induces an increase in phospholipase C, leading to activation of downstream proteins such as MAPK [25], Janus kinases and STAT (signal transducers and activators of transcription) proteins [26]. More recently, a study by Greco and colleagues [27] conducted in MCF-7 cells and primary breast cancer cells revealed that AT1 receptor regulates mitogenic signalling pathways by two simultaneous mechanisms, one involving protein kinase C and the other EGFR transactivation [27].

We conducted the present study to investigate a combined rapid oestrogen and epidermal growth factor (EGF) activation of the MAPK cascade in both ER-positive and ER-negative breast cancer cells. We describe a role for AT1 in mediating this nongenomic oestrogen-signalling pathway.

## Materials and methods

### Cell lines and primary cell cultures

Human breast cancer cell lines MCF-7 (ER-positive) and SKBR3 (ER-negative) were obtained from American Type Culture Collection (Rockville, MD, USA) and cultured in RPMI 1640 medium (Sigma Ltd, Ireland), enriched with 5% foetal bovine serum (Sigma Ltd) and supplemented with the antibiotics penicillin (100 ng/ml) and streptomycin (100 ng/ml; Invitrogen Ltd, UK) and amphotericin B (2 μg/ml; Invitrogen Ltd, Paisley, UK).

Primary cell cultures derived from histologically confirmed ER-positive and ER-negative breast primary tumours were prepared as previously described [28]. In brief, following ethical approval breast tumour specimens were obtained from patients undergoing surgery for removal of a histologically confirmed breast tumour. Primary tumour epithelial cells were extracted in Hanks' balanced salt solution without calcium or magnesium (Gibco, Paisley, Scotland) supplemented with 1 μmol/l EDTA and 1 μmol/l DTT for 40 minutes. Cells were cultured in RPMI containing 5 μg/ml insulin, 10 μg/ml transferrin, 30 nmol/l sodium selinate, 10 nmol/l hydrocortisone, 10 nmol/l oestradiol, 10 mmol/l Hepes, 2 mmol/l glutamine, 10% foetal calf serum (weight/vol) and 5% ultroser G on a growth factor reduced matrigel matrix (60 ng/cm^2^; BD Biosciences, San Jose, CA). Cell viability and epithelial origin of tumour cells were confirmed as previously described [28].

Human breast cancer cells were incubated in a humidified atmosphere of 5% carbon dioxide at 37°C. Experiments were carried out when the cells reached 90% confluence and following 24 hours incubation in serum-free medium without phenol red. Where indicated, cells were preincubated with receptor antagonists 60 minutes before addition of EGF (Sigma) and/or 17 μ-oestradiol (10^-8 ^mol/l; Sigma). EGFRs were inhibited with tyrophostin AG1478 (150 nmol/l), GCPRs with pertussis toxin (50 ng/ml), and angioteinsin II receptors with saralasin A2275 (1 μmol/l; Sigma).

### MTT thiozolyl blue proliferation assay

Cell proliferation was measured using MTT thiazolyl blue assay. Approximately 1,000 cells were seeded in each well of a 96-well plate, cultured, serum starved without phenol red and treated as above. Then, 5 mg/ml MTT (Sigma) in 1:10 dilution per well was added and incubated for 3–4 hours. The cells were lysed by adding 200 μl/well of dimethyl sulfoxide (Sigma Ltd) and read at 570 nm absorbance wavelength in a microtitre plate spectrophotometer.

### Western blot analysis

Samples containing 50 μg protein were electrophoresed on 12% SDS polyacrylamide gels (Bio-Rad Laboratories Ltd, Hertfordshire, UK) and transferred onto a nitrocellulose membrane. The membranes were probed with the phospho-Raf, phospho-ERK (extracellular signal regulated kinase)1/2 (1:1000; Cell Signalling), Raf, ERK1/2 and AT1 (1 μg/ml; Santa Cruz, CA, USA) followed by the corresponding horseradish peroxidase-conjugated mouse or rabbit secondary antibodies (Amersham Biosciences, Buckinghamshire, UK). Chemiluminescence detections were carried out using Luminol (Santa Cruz) or enhanced chemiluminescence with SuperSignal (Pierce, Rockford, IL, USA).

### cAMP ELISAs

Concentrations of cAMP were measured using cAMP enzyme immunoassay in accordance with the manufacturer's protocol (Sigma-Aldrich Ireland Ltd, Dublin, Ireland). The assays were performed in a 96-well plate coated with anti-rabbit IgG antibody. The coloured end products, produced by addition of substrate to the wells, were read at 405 nm absorbance wavelength on a multiwell plate reader. The intensity of the colour was inversely proportional to the concentration of cAMP present in the well.

### Immunodetection/microscopy

Immunodetection was carried out on fixed SKBR3 breast cancer cells and primary breast cancer tissue. Immunocytochemistry and immunohistochemistry were used to detect phospho-ERK1/2 and AT1 on fixed cells and on paraffin-embedded tissue, respectively. SKBR3 cells were cultured on 8-well chamber slides (Lab Tek, Campbell, CA, USA) and subsequently fixed and permeabilized. For tissue, 5 μm sections were cut from paraffin-embedded breast tumour blocks and mounted on slides. Sections were dewaxed and rehydrated. Antigen retrieval was performed by immersing section in 0.6 mol/l citrate buffer and microwaving on high for 7 minutes. Endogenous peroxidase activity was blocked in fixed cells and tumour sections with 3% hydrogen peroxide for 20 minutes. Antigens were detected using the Vectastain Elite kit (Vectra Labs, Burlingame, CA, USA) in accordance with the manufacturer's instructions. Briefly, cells and sections were blocked in serum for 90 minutes. Cells and sections were incubated with primary antibodies, mouse anti-human phospho-ERK 1/2 [1:400]; New England Biolabs, Ipswitch, MA, USA) or rabbit anti-human AT1 receptor (2 μg/ml; Santa Cruz) for 60 minutes at room temperature. Subsequently, sections were incubated in the corresponding biotin-labelled secondary antibody (1 in 2000) for 30 minutes, followed by peroxidase-labelled avidin biotin complex. Sections were developed in 3,3-diaminobenzidine tetrahydrochloride and counterstained with haematoxylin. Negative controls were performed using matched IgG controls (Dako, Glostrup, Denmark). Sections were examined under a light microscope.

Immunofluorescence detection of AT1 was performed on SKBR3 cells and primary breast cancer tissue. Cells and tissue were prepared as described above. Breast cancer cells and sections were blocked in 1.5% normal serum and then incubated with 20 μg/ml rabbit anti-human AT1 in 10% human serum for 90 minutes. Cells and sections were subsequently incubated with Alexa Fluor 594-conjugated secondary antibody (Molecular Probes, Paisley, UK) for 60 minutes and were counterstained with DAPI (Sigma-Aldrich). Confocal microscopy was performed using a confocal microscope (model MRC 1024; Bio-Rad Laboratories, Hertfordshire, UK) and images were captured using Laser Capture software (Zeiss LSM 510 UV META system, Carl Zeiss Ltd., Hertfordshire, UK).

### Small interfering RNA transfection

Small interfering RNAs (siRNAs) predesigned by Ambion (Austin, TX, USA) were used. The siRNA sequences targeting AT1 (AT1 #1 [AGTR1 siRNA ID:1988], 5'-GGCCCUAAAGAAGGCUUAUtt-3' [NM_000685]; AT1 #2 [AGTR1 siRNA ID:2083], 5'-GGCUUAUGAAAUUCAGAAGtt-3' [NM_000685]) were assessed for their ability to downregulate AT1 protein expression. Silencer Negative Control #1 (scrambled) siRNA or Silencer^® ^GAPDH siRNA (Human; Ambion) were used in accordance with the manufacturer's instructions as control siRNA. SKBR3 cells were transfected using a Nucleofector (Amaxa Biosystems, Cologne, Germany). Cells were transfected with 1.5 μg siRNA in Nucleofector solution V using programme A-23. Immediately following tranfection RPMI was added to the SKBR3 cells, which were then plated in 6-well tissue culture plates overnight. Cells were collected the following day and analyzed for protein expression by Western blotting.

### Statistical analysis

The strengths of associations between the various parameters measured during this study were tested using nonparametric tests, which do not assume that data are normally distributed. Mann-Whitney *U *test was used to compare two independent groups of sampled data, analyzed by Statview^® ^statistical program (SAS, Cary, NC, USA). Two-sided *P *values below 0.05 were considered statistically significant. Data are expressed as mean ± standard error of the mean.

## Results

### 17β-Oestradiol and EGF alone and in combination induced breast cancer cell proliferation and rapid activation of the MAPK pathway

**Figure 1:**
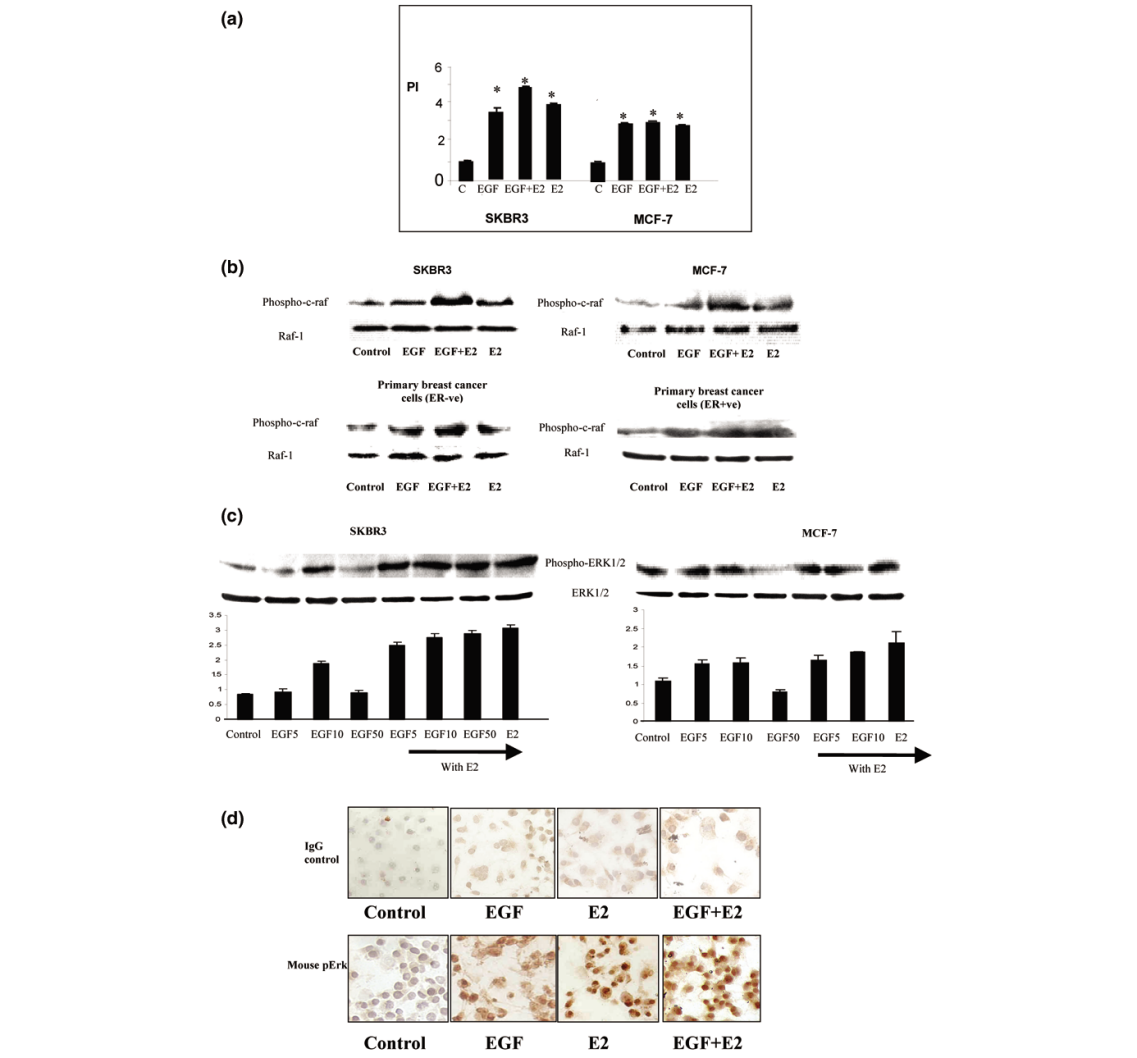
Effect of 17β-oestradiol and EGF on cell proliferation and induction of MAPK protein expression in breast cancer cells. **(a) **Cell proliferation findings. SKBR3 and MCF-7 human breast cancer cells, and ER-negative and ER-positive primary breast cultures derived from patient tumours were treated with EGF (10 ng/ml) and 17β-oestradiol (10^-8 ^mol/l) alone and in combination for 24 hours. Cell proliferation assays were carried out using MTT thiozolyl blue. Proliferative index of the control group is standardised to 1. The results shown are expressed as mean ± standard error (*n *= 9). Statistical analysis was performed using Mann Whitney *U* test (*P < 0.02 versus control). **(b) **Western blot analysis of phospho-Raf and total Raf protein expression. SKBR3 and MCF-7 human breast cancer cells, and ER-negative and ER-positive primary breast cultures derived from patient tumours were treated with EGF (10 ng/ml) and 17β-oestradiol (10^-8 ^mol/l) alone and in combination for 10 minutes. **(c) **Phospo-ERK1/2 and total ERK1/2 protein expression. SKBR3 and MCF-7 human breast cancer cells were treated with 5, 10 and 50 ng/ml EGF and 17β-oestradiol (10^-8 ^mol/l) alone and in combination for 10 minutes. Results are representative of those obtained in three separate experiments. **(d) **Immunocytochemical localisation of phospho-ERK in ER-negative SKBR3 cells treated with EGF (10 ng/ml) and 17β-oestradiol (10^-8 ^mol/l) alone and in combination for 30 minutes. The slides were counter stained with Mayer's haematoxylin solution and viewed under a light microscope at 200× magnification. Positive cells stained cells stained brown against a blue background. Results are representative of those obtained in three separate experiments. E2, 17β-oestradiol; EGF, epidermal growth factor; ER, oestrogen receptor; ERK, extracellular signal regulated kinase; MAPK, mitogen-activated protein kinase; PI, proliferative index.

Both 17β-oestradiol and EGF induced cell proliferation in ER-negative SKBR3 cells and ER-positive MCF-7 cells (Figure 1a). In SKBR3 cells, combined treatment with 17β-oestradiol and EGF induced a further increase in cell proliferation compared with either treatment alone. To examine the effect of 17β-oestradiol and growth factor treatment on MAPK activation, we examined their ability to induce phosphorylation of Raf and ERK1/2. In ER-positive and ER-negative breast cancer cell lines and in primary cell cultures derived from patient tumours, both 17β-oestradiol and EGF increased expression of phospho-Raf and phospho-ERK1/2 (Figure 1b, c). Combined treatment with steroid and growth factors resulted in a further increase in phosphorylation of the MAPK proteins. The ability of 17β-oestradiol and EGF to mobilize ERK1/2 was also examined. Increased nuclear localization of phospho-ERK1/2 was observed in the presence of EGF and in particular in the presence of 17β-oestradiol and 17β-oestradiol in combination with EGF (Figure 1d).

### Rapid estrogen signalling is dependent on tyrosine kinase receptors

**Figure 2:**
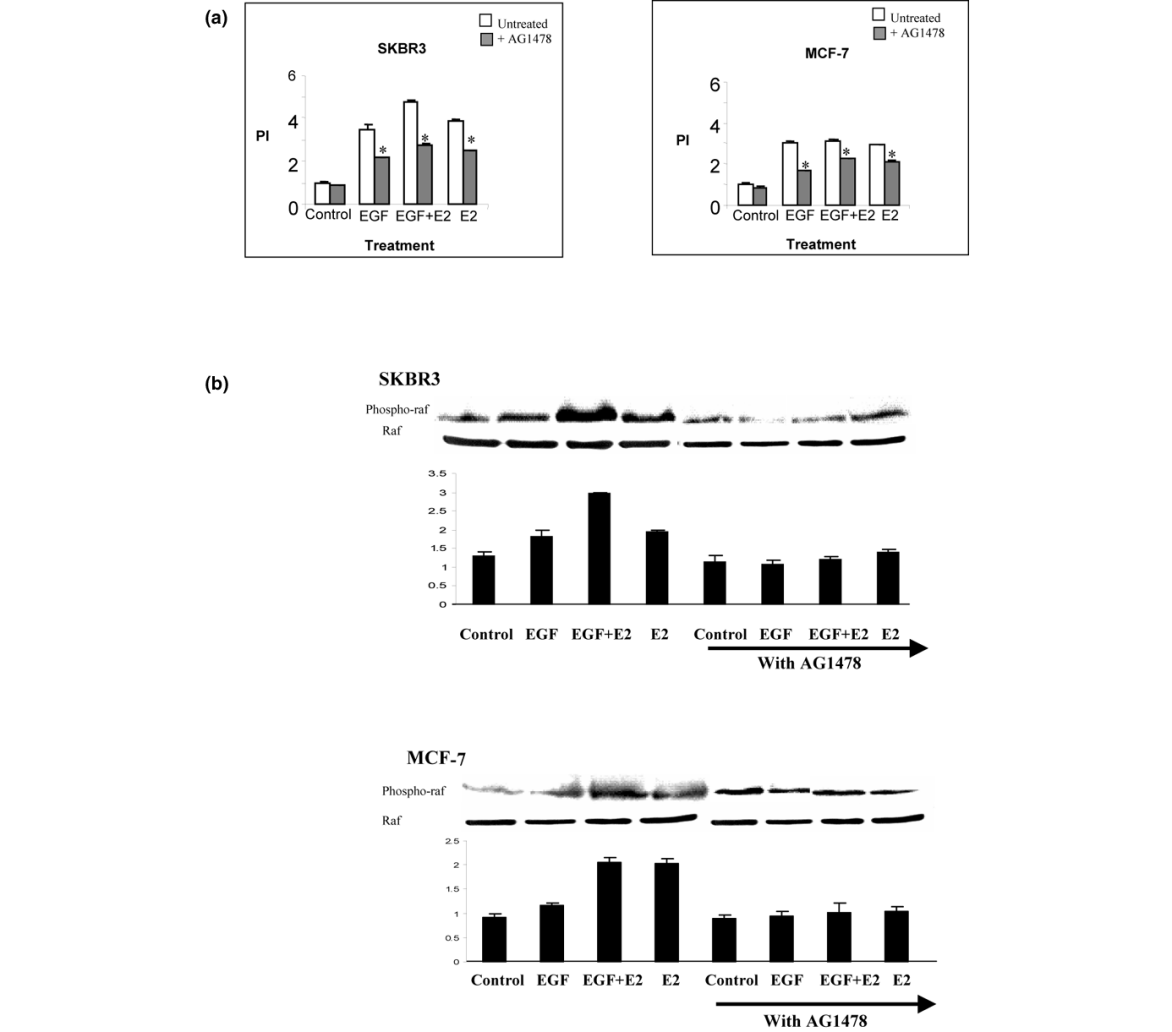
17β-estradiol and EGF induced cell proliferation and raf phosphorylation is mediated through EGFR. **(a) **SKBR3 and MCF-7 breast cancer cells were pretreated with or without the EGFR antagonist AG1478 (150 nmol/l) 1 hour before treatment with EGF (10 ng/ml) and 17β-oestradiol (10^-8 ^mol/l) alone and in combination for 24 hours. Cell proliferation assays were carried out using MTT thiozolyl blue. Proliferative index of the control group is standardized to 1. The results shown are expressed as mean ± standard error (*n *= 9). Statistical analysis was performed using Mann-Whitney *U* test (*P < 0.02 versus without AG1478). **(b) **Western blot analysis of phospho-Raf and total Raf in SKBR3 and MCF-7 breast cancer cells pretreated with or without the EGFR antagonist AG1478 (150 nmol/l) for 1 hour before 10 minutes of incubation with EGF (10 ng/ml) and 17β-oestradiol (10^-8 ^mol/l) alone and in combination. Results are representative of those obtained in three separate experiments. E2, 17β-oestradiol; EGFR, epidermal growth factor receptor; PI, proliferative index.

It has been reported that oestrogen transactivates the EGFR to initiate the MAPK cascade. To examine the role of tyrosine kinase receptor EGFR in mediating 17β-oestradiol-induced cell proliferation and MAPK activation in ER-positive and ER-negative breast cancer cells, we inhibited EGFR with the specific inhibitor AG1478. 17β-Oestradiol and EGF induced cell proliferation was attenuated in the presence of AG1478 (Figure 2a). The EGFR antagonist also diminished steroid and growth factor induced Raf phosphorylation in both SKBR3 and MCF-7 breast cancer cell lines (Figure 2b).

### Oestrogen can signal through G proteins in ER-positive and ER-negative breast cancer cell lines

**Figure 3:**
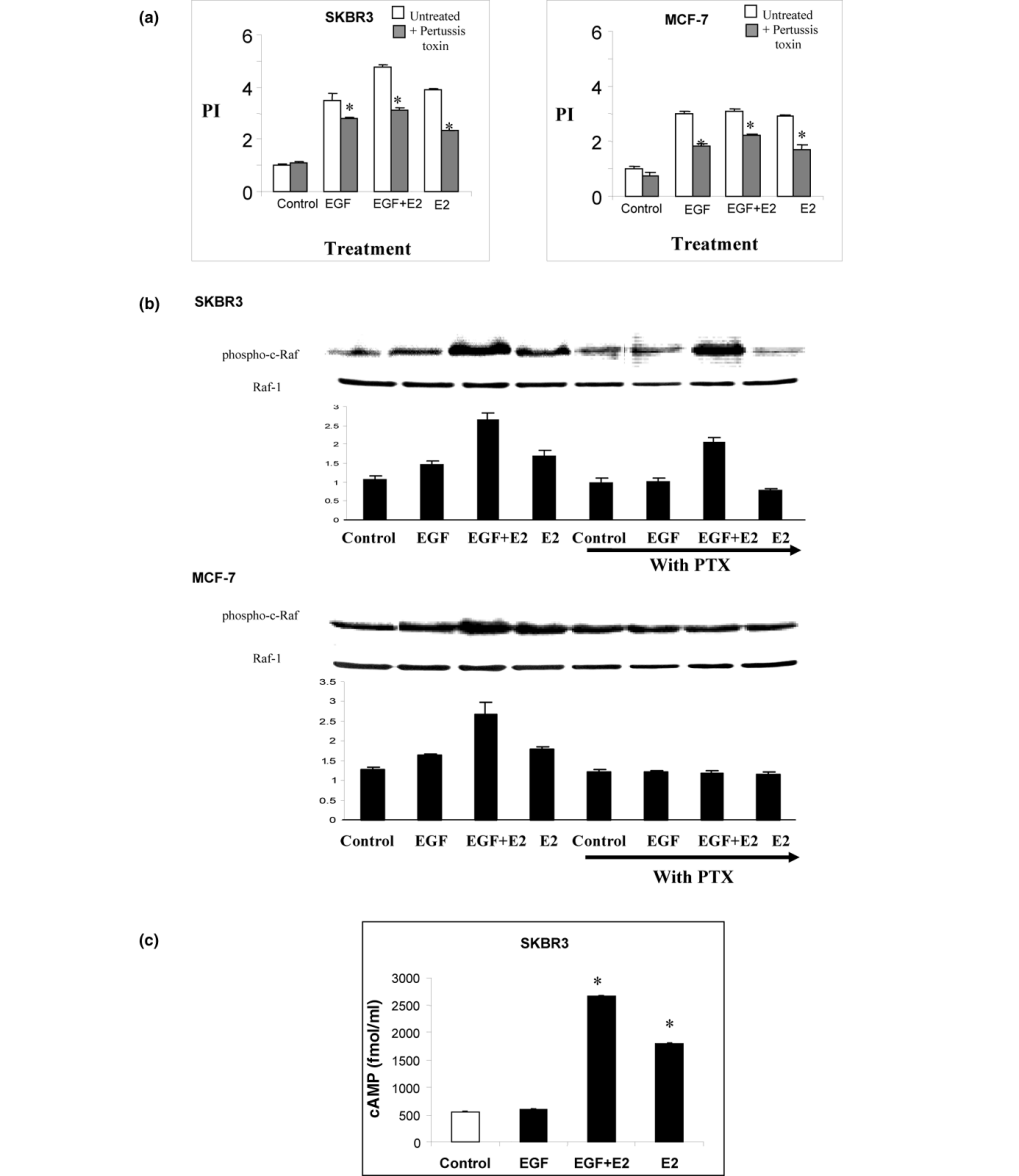
Effect of GPCR antagonism on 17β-oestradiol and EGF induced cell proliferation, Raf phosphorylation and cAMP production in breast cancer cells. **(a) **SKBR3 and MCF-7 breast cancer cells pretreated with or without the GPCR antagonist PTX (50 ng/ml) 1 hour before 24 hours of incubation EGF (10 ng/ml) and 17β-oestradiol (10^-8 ^mol/l) alone and in combination. Cell proliferation assays were carried out using MTT thiozolyl blue. Proliferative index of the control group is standardized to 1. The results shown are expressed as mean ± standard error (*n *= 9). Statistical analysis was performed using Mann Whitney *U* test (*P < 0.02 versus without PTX). **(b) **Western blot analysis of phospho-Raf and total Raf in SKBR3 and MCF-7 cells pretreated with or without the GPCR antagonist pertussis toxin (50 ng/ml) for 1 hour before 10 minutes of incubation with EGF (10 ng/ml) and 17β-oestradiol (10^-8 ^mol/l) alone and in combination. Results are representative of those obtained in three separate experiments. **(c) **Intracellular cAMP levels in SKBR3 cells treated with EGF (10 ng/ml) and 17β-oestradiol (10^-8 ^mol/l) alone and in combination. Results are expressed as mean ± standard error (*n *= 9). Statistical analysis was performed using Mann-Whitney *U* test (*P < 0.02 versus control). E2, 17β-oestradiol; EGFR, epidermal growth factor receptor; GPCR, G-protein-coupled receptor; PI, proliferative index.

It has been suggested that oestrogens can activate either membrane-bound ER or GPCR to initiate rapid cell signalling events. We examined the role of G proteins in 17β-oestradiol and EGF-induced cell phosphorylation and activation of the MAPK pathway, in ER-positive and ER-negative cell lines. The G protein antagonist pertussis toxin inhibited 17β-oestradiol cell growth and Raf phosphorylation in both ER-positive and ER-negative cell lines (Figure 3a, b). Of interest, treatment with pertussis toxin also abrogated the cell growth and Raf phosphorylation seen in the presence of EGF and EGF in combination with 17β-oestradiol, in particular in ER-positive MCF-7 breast cancer cells (Figure 3a, b). We assessed the ability of 17β-oestradiol and EGF to induce the classic GPCR second messenger cAMP. In SKBR3 cells, levels of cAMP were increased in the presence of 17β-oestradiol and in the presence of 17β-oestradiol in combination with EGF (Figure 3c).

### Oestrogens can signal through the angiotensin II receptor AT1 in human breast cancer cells

**Figure 4:**
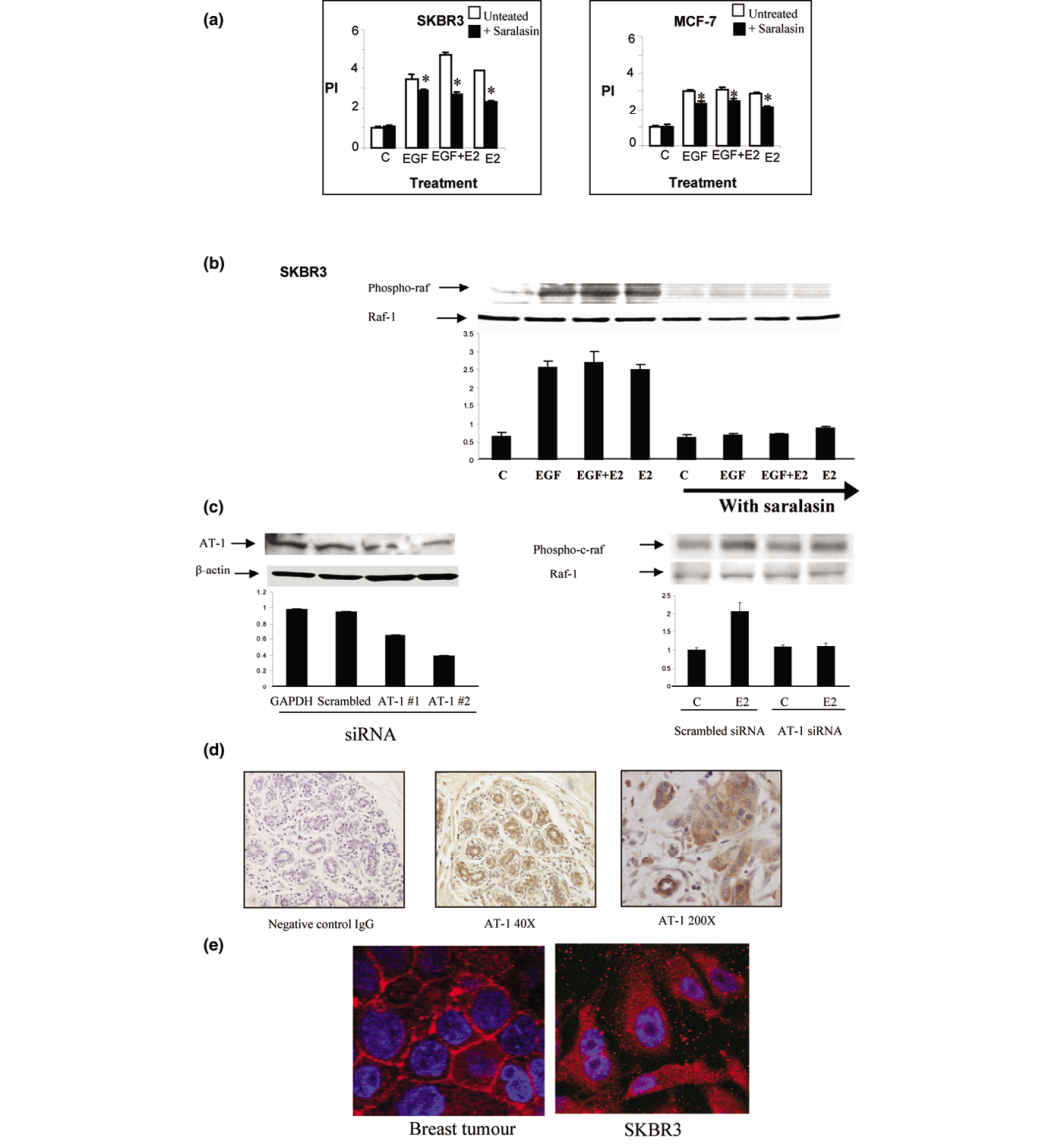
The role of the AT1 receptor in 17β-oestradiol and EGF-mediated cell proliferation and Raf phosphorylation in breast cancer cells. **(a) **SKBR3 and MCF-7 breast cancer cells pretreated with or without angiotensin II receptor antagonist saralasin (10^-6 ^mol/l) 1 hour before 24 hours of incubation with EGF (10 ng/ml) and 17β-oestradiol (10^-8 ^mol/l) alone and in combination. Cell proliferation assays were carried out using MTT thiozolyl blue. Proliferative index of the control group is standardized to 1. The results shown are expressed as mean ± standard error (*n *= 9). Statistical analysis was performed using Mann-Whitney *U* test (*P < 0.02 versus without saralasin). **(b) **Western blot analysis of phospho-Raf and total Raf in SKBR3 cells pretreated with or without the AT1 antagonist saralasin (10^-6 ^mol/l) for 1 hour before 10 minutes of incubation with EGF (10 ng/ml) and 17β-oestradiol (10^-8 ^mol/l) alone and in combination. Results are representative of those obtained in three separate experiments. **(c) **Western bolt analysis of AT1 and phospho-Raf in SKRB3 cells transiently transfected with GAPDH siRNA, scrambled siRNA, or AT1 (#1 or #2) siRNA before treatment with 17β-oestradiol (10^-8 ^mol/l) for 10 minutes. Results are representative of those obtained in three separate experiments. Optical density readings of control values were normalized to 1 and experimental groups were expressed as a ratio. Values are expressed as mean ± standard error (*n *= 3). **(d) **Immunohistochemistry was carried out for AT1 receptor on primary human breast cancer tissue (7 μm). Negative control was matched IgG. The slides were counterstained with Mayer's haematoxylin solution and were viewed under a light microscope at 40× and 200× magnifications. Positive cells stained cells stained brown against a blue background. **(e) **Immunofluorescence studies of AT1 receptor in human primary tumour breast cancer tissue (7 μm) and SKBR3 breast cancer cells. Slides were viewed under a confocal microscope at 630×. AT1, angiotensin II type 1; E2, 17β-oestradiol; EGFR, epidermal growth factor receptor; siRNA, small interfering RNA.

A role for the GPCR AT1 was assessed in ER-positive and ER-negative breast cancer cells. Treatment with the AT1 receptor antagonist saralasin resulted in attenuation of 17β-oestradiol and EGF induced cell proliferation in the ER-negative SKBR3 cells and, to a lesser extent, in the ER-positive MCF-7 cells (Figure 4a). In the SKBR3 cells saralasin inhibited 17β-oestradiol induced phosphorylation of Raf (Figure 4b). To investigate further the role of the AT1 receptor, we knocked down AT1 using siRNA technology. Two predesigned siRNA sequences targeting AT1 (AT1 #1 and AT1 #2) were assessed for their ability to knock down AT1 protein expression, compared with siRNA sequences targeting GAPDH and scrambled siRNA. AT1 #2 siRNA was found to be more effective at downregulating AT1 protein expression and was therefore used in subsequent experiments (Figure 4c). The ability of 17β-oestradiol to induce Raf phosphorylation in SKBR3 cells was attenuated in cells transfected with AT1 #2 siRNA compared with scrambled control (Figure 4c).

In paraffin-embedded breast cancer tissue AT1 protein was found to be expressed predominantly in breast tumour epithelial cells, with little staining detected in the surrounding stromal cells (Figure 4d). In order to determine cellular localization of AT1 we performed confocal microscopy. AT1 was found to be expressed predominantly at the cellular membrane in tumour epithelial cells of breast cancer tissue and in the SKBR3 breast cancer cell line (Figure 4e).

## Discussion

The ability of oestrogen to transactivate EGFRs rapidly in a G-coupled protein dependent manner has now been established. The mechanism of this 'nongenomic' oestrogen signalling and its dependence on a membrane-bound ER, however, remains controversial. Studies have shown that membrane ER is similar if not identical to nuclear ER [16], which is linked to G proteins, activating several second messenger systems [29]. Investigations using ER-negative cell lines have demonstrated that oestrogen may also function through ER-independent mechanisms. GPCRs, and in particular GPR30, the orphan GPCR, have been implicated in mediating this ER-independent oestrogen signalling [21,22]. In this study we examined rapid oestrogen signalling in ER-positive and ER-negative, breast cancer cell lines and primary breast cancer cells derived from patient tumours and investigated a role for the GPCR AT1 in mediating this effect.

Nongenomic actions of oestrogen result in an array of downstream signalling events, which are thought to be largely cell specific. In breast cancer, rapid oestrogen events have been shown to include accumulation of cAMP, ERK1/2 and c-fos [22,30]. In these studies 17β-oestradiol and EGF induced increased cell proliferation in both ER-positive MCF-7 and ER-negative SKBR3 breast cancer cell lines. We examined the ability of 17β-oestradiol and EGF alone and in combination to activate the MAPK cascade. In breast cancer cell lines and in primary breast tumour cell cultures, expression of ER was not required for 17β-oestradiol induced phosphorylation of Raf. Furthermore, in line with other investigators who have described activation of ERK1/2 in ER-negative cells [21], we found that 17β-oestradiol induced ERK1/2 phosphorylation and translocation from the cytosol to the nucleus in SKBR3 cells. The ability of oestrogens to initiate the MAPK cascade has been linked to Gβγ protein dependent release of surface associated heparin-binding EGF, resulting in transactivation of the EGFR [21,23]. Here, requirement of EGFR transactivation for maximal oestrogen-mediated cell proliferation and MAPK activation was established using the receptor EGF inhibitor AG1478.

Both ER-dependent and ER-independent transactivation of EGFR has been shown to signal via G coupled proteins, with several different G protein heterodimers coupling with the same receptor. Membrane ER can co-immunoprecipitate with Gs and Gq proteins in transfected and endogenous ER cell models [16,19], and in ER-negative cells oestrogen-GPR30 dependent activation of MAPK is sensitive to the Gi/o-protein inhibitor pertussis toxin [22]. Here, pertussis toxin attenuated 17β-oestradiol induced cell proliferation and Raf phosphorylation in both ER-positive and ER-negative breast cancer cell lines. Of interest, pertussis toxin also attenuated EGF-induced breast cancer cell proliferation and phospho-Raf expression. These observations are consistent with those of other investigators that have observed pertussis toxin induced reductions in growth factor mediated ERK1/2 activation [31,32]. It has been proposed that these effects may be mediated via pertussis toxin induced disinhibition of cAMP [31]. To assess further the role of G coupled proteins we evaluated the accumulation of the GPCR second messenger cAMP, in response to both 17β-oestradiol and EGF. As previously reported 17β-oestradiol induced cAMP levels in ER negative SKBR3 breast cancer cells [23]. Although EGF alone had no effect on cAMP accumulation, EGF synergistically increased oestrogen-induced cAMP, providing further evidence of crosstalk between tyrosine kinase receptors and G proteins.

Mediation of the nongenomic effects of oestrogens are likely to occur in a cell-specific manner, with more than one GPCR participating in rapid oestrogen signalling. In addition to GPR30, the membrane bound sex hormone binding globulin receptor can mediate oestrogen-induced activation of adenylate cyclase via the Gs protein subunit. The angiotensin II receptor AT1 is another attractive oestrogen-signalling GPCR candidate. AT1 is a Gq/i coupled receptor, which can lead to tyrosine kinase phosphorylation, increased phospholipase C [25,26] and Raf-ERK1/2 activation, which can be partially inhibited by pertussis toxin [33]. Expression of AT1 in normal and diseased breast tissue has previously been reported [24]. In the present study immunohistochemistry performed on primary breast cancer tissue revealed AT1 receptor staining primarily in breast tumour epithelial cells. At a cellular level AT1 was found to be predominantly expressed in the membrane of tumour epithelial cells and ER-negative breast cancer cell lines. Here, we investigated the role of the AT1 in mediating the nongenomic effects of oestrogens in ER-positive and ER-negative breast cancer cells. The angiotensin II receptor competitive inhibitor saralasin attenuated the proliferative effects of 17β-oestradiol and EGF in SKBR3 and MCF-7 breast cancer cells, in a similar manner to that seen for pertussis toxin. Of interest, the inhibitory effects of saralasin were found to be greater in the ER-negative cells than in ER-positive cells, which is consistent with the proposed cell specific nature of nongenomic estrogen signalling. Furthermore, 17β-oestradiol mediated Raf phosphorylation was inhibited in the presence of saralasin in SKBR3 cells. To confirm a role for AT1 in nongenomic oestrogen signalling in ER-negative cells, we knocked down AT1 expression with siRNA. Downregulation of AT1 also attenuated 17β-oestradiol induction of phospho-Raf in the ER-negative SKBR3 cells.

## Conclusion

The mechanisms by which oestrogen couples to G proteins to mediate its nongenomic effects are likely to be varied and cell context specific. The data presented here indicate that estrogens can activate early cell survival signalling in an ER-independent manner not only in ER-negative cell lines but also in primary breast cultures. We propose that this ER-independent oestrogen signalling is mediated, at least in part, through the GPCR AT1. These data suggest that in a clinical setting aromatase inhibitors may be beneficial in treating ER-negative as well as ER-positive breast tumours. Elucidation of the components of the nongenomic oestrogen signalling cascade will provide important information regarding the role of oestrogens in physiological and pathophysiological conditions.

## Abbreviations

AT1 = angiotensin II type 1 receptor; EGF = epidermal growth factor; EGFR = epidermal growth factor receptor; ER = oestrogen receptor; ERK = extracellular signal-regulated kinase; GPCR = G-protein-coupled receptor; MAPK = mitogen-activated protein kinase; siRNA = small interfering RNA.

## Competing interests

The authors declare that they have no competing interests.

## Authors' contributions

KL performed cell cultures, proliferation assays, Western blot analysis and ELISA studies. NC completed immunofluorescence and knockdown studies. AH, supplied tumour specimens and provided clinical interpretation of data. LY contributed to conception and design of study and interpretation of data. All authors read and approved the final manuscript.
