# Correction to: Nongenomic oestrogen signalling in oestrogen receptor negative breast cancer cells: a role for the angiotensin II receptor AT1

**DOI:** 10.1186/s13058-018-0987-x

**Published:** 2018-06-20

**Authors:** Kheng Tian Lim, Niamh Cosgrave, Arnold David Hill, Leonie S. Young

**Affiliations:** 1 School of Medicine and Medical Science, St. Vincent’s University Hospital, Elm Park, Dublin, Ireland; 2 School of Medicine and Medical Science, UCD Conway Institute of Biomolecular and Biomedical Research, UCD Conway Institute, University College Dublin, Dublin, Ireland

## Correction

After the publication of this work [1] errors were noticed in the total protein loading controls for Figs. 1C, 2B, 3B and 4B. These errors do not affect the interpretation of the data. The corrected figures are shown below. We apologize for this error.

**Fig. 1:**
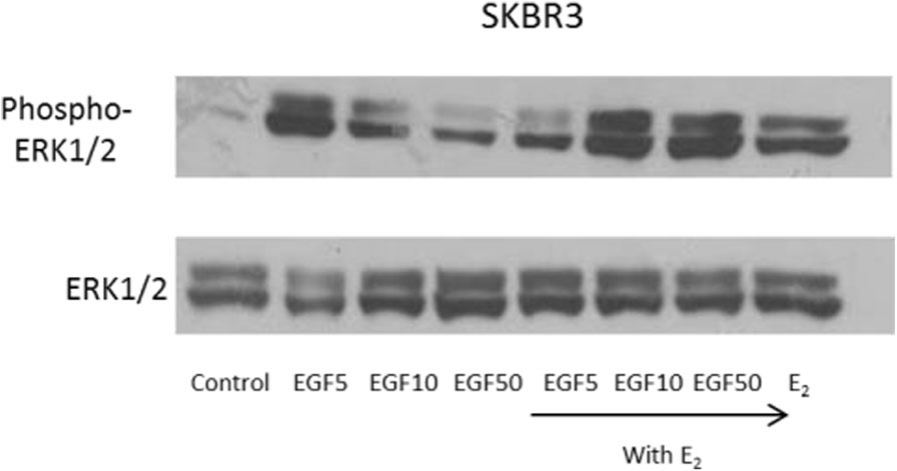
Effect of 17β-oestradiol and EGF on cell proliferation and induction of MAPK protein expression in breast cancer cells. **(c)** SKBR3 breast cancer cells were treated with 5, 10 and 50 ng/ml EGF and 17β-oestradiol (10^− 8^ mol/l) alone and in combination for 10 min. 40 μg protein was electrophoresed on a 10% gel and transferred to nitrocellulose. The membrane was probed with rabbit anti-Phospho-Erk1/2 antibody (Thr202/Tyr204 - Cell Signalling # 4370) and mouse anti-Erk1/2 antibody (Cell Signalling # 4696)

**Fig. 2:**
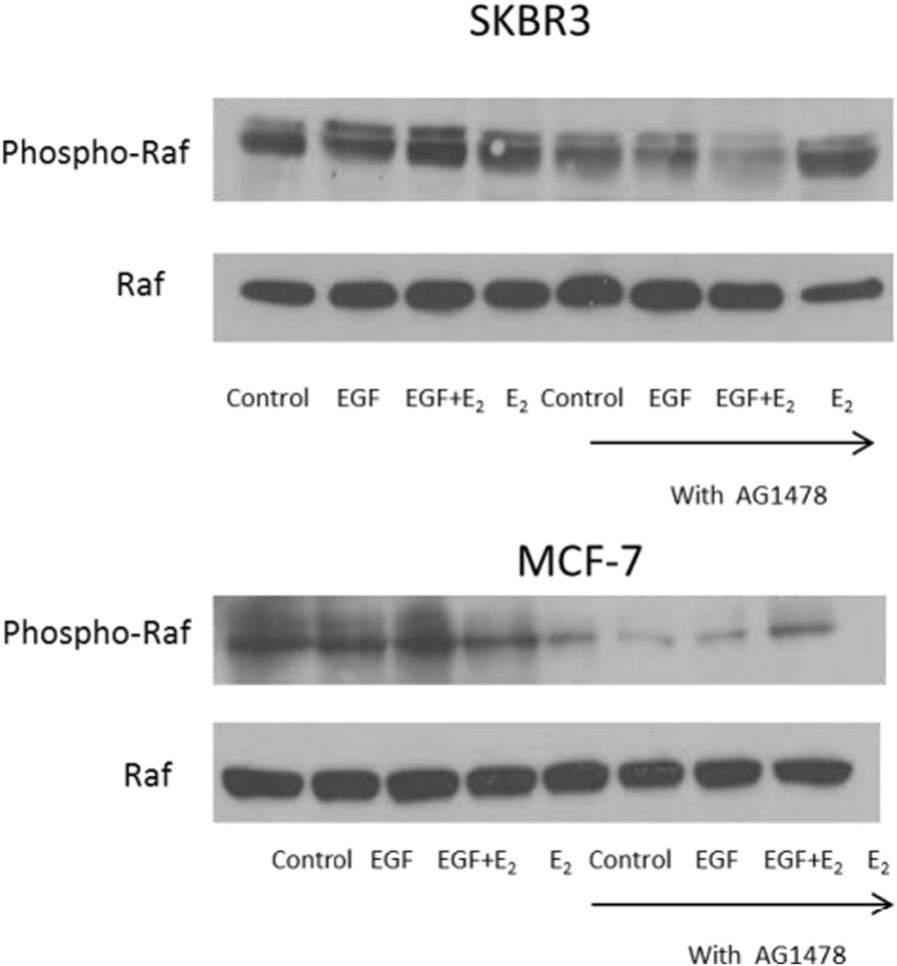
17β-oestradiol and EGF induced cell proliferation and raf phosporylation is mediated through EGFR. **(b)** SKBR3 and MCF-7 breast cancer cells were pre-treated with the EGFR antagonist AG1478 (150 nmol/l) for 1 h before 10 min of incubation with EGF (10 ng/ml) and 17β-oestradiol (10^− 8^ mol/l) alone and in combination. 40 μg protein was electrophoresed on a 10% gel and transferred to nitrocellulose. The membrane was probed with rabbit anti-Phospho-Raf antibody (Ser259 - Cell Signalling # 9421) and mouse anti-Raf antibody (Santa Cruz sc-373,722)

**Fig. 3:**
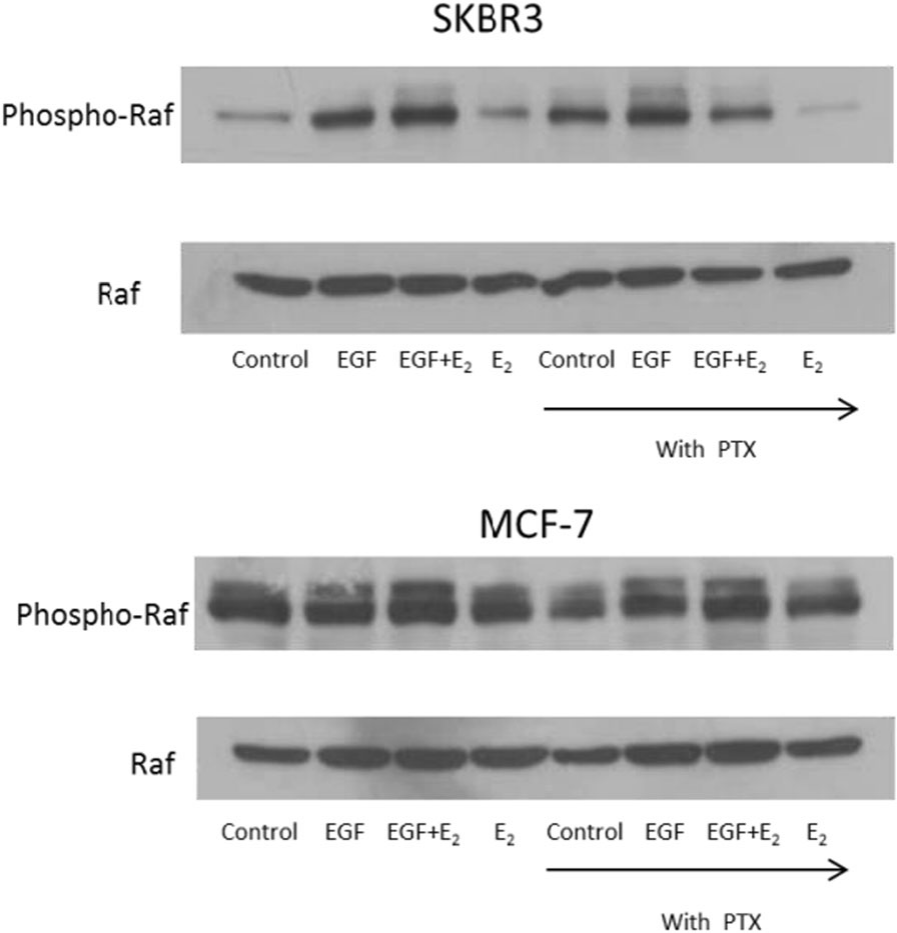
Effect of GPCR antagonism on 17β-oestradiol and EGF induced cell proliferation, raf phosporylation and cAMP production in breast cancer cells. **(b)** SKBR3 and MCF-7 cells were pre-treated with the GPCR antagonist pertussis toxin (50 ng/ml) for 1 h before 10 min of incubation with EGF (10 ng/ml) and 17β-oestradiol (10^− 8^ mol/l) alone and in combination. 40 μg protein was electrophoresed on a 10% gel and transferred to nitrocellulose. The membrane was probed with rabbit anti-Phospho-Raf antibody (Ser259 - Cell Signalling # 9421) and mouse anti-Raf antibody (Santa Cruz sc-373,722)

**Fig. 4:**
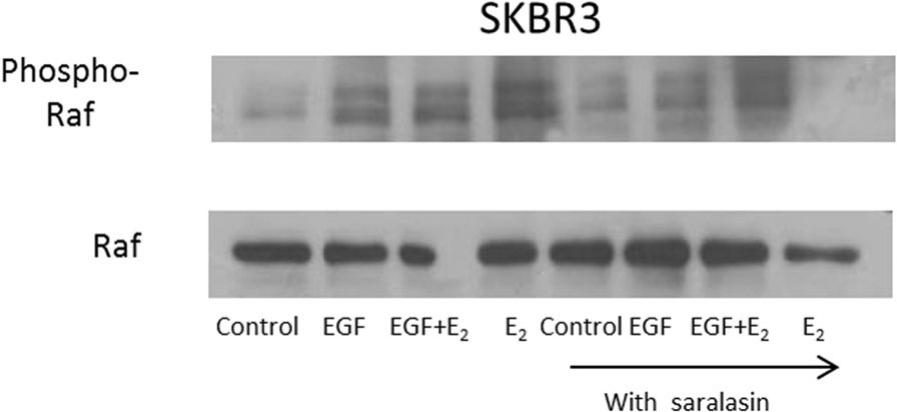
*The role of the AT1 receptor in* 17β-oestradiol and EGF mediated cell proliferation and raf phosphorylation breast cancer cells. **(b)** SKBR3 cells were pre-treated with the AT1 antagonist saralasin (10^− 6^ mol/l) for 1 h before 10 min of incubation with EGF (10 ng/ml) and 17β-oestradiol (10^− 8^ mol/l) alone and in combination. 40 μg protein was electrophoresed on a 10% gel and transferred to nitrocellulose. The membrane was probed with rabbit anti-Phospho-Raf antibody (Ser259 - Cell Signalling # 9421) and mouse anti-Raf antibody (Santa Cruz sc-373,722)
